# Association between methylphenidate use and long-term cardiovascular risk in paediatric patients with attention deficit and hyperactivity disorder

**DOI:** 10.1136/bmjpo-2024-002753

**Published:** 2024-09-03

**Authors:** Heng-Ching Liao, Chien-Ning Hsu, Fang-Ju Lin, Susan Shur-Fen Gau, Chi-Chuan Wang

**Affiliations:** 1 Graduate Institute of Clinical Pharmacy, National Taiwan University, Taipei, Taiwan; 2 Department of Pharmacy, National Taiwan University Cancer Center, Taipei, Taiwan; 3 Department of Pharmacy, Chang Gung Memorial Hospital Kaohsiung Branch, Kaohsiung, Taiwan; 4 School of Pharmacy, Kaohsiung Medical University, Kaohsiung, Taiwan; 5 Department of Pharmacy, National Taiwan University Hospital, Taipei, Taiwan; 6 School of Pharmacy, National Taiwan University, Taipei, Taiwan; 7 Department of Psychiatry, National Taiwan University Hospital, Taipei, Taiwan; 8 College of Medicine, National Taiwan University, Taipei, Taiwan

**Keywords:** Child Psychiatry, Noncommunicable Diseases, Statistics, Therapeutics

## Abstract

**Background:**

There have been concerns about the potential cardiovascular (CV) adverse effects associated with methylphenidate (MTH) use. However, only limited evidence exists on the long-term safety of MTH.

**Objective:**

To evaluate whether MTH use is associated with long-term CV risk.

**Methods:**

This was a retrospective cohort study using 2003–2017 data from the Health and Welfare Database in Taiwan. Patients newly diagnosed with attention deficit and hyperactivity disorder (ADHD) and between 3 and 18 years of age were included. Two treatment statuses were assessed: initial treatment ≥7 days and ≥180 days. Patients treated with MTH were compared with those receiving non-medication therapy. One-to-one propensity score matching was used to balance between-group differences. Study outcomes included major CV events, chronic CV disease, cardiogenic shock and all-cause mortality. Cox proportional hazard models were used to estimate HRs between the two groups.

**Results:**

We began with 307 459 patients with ADHD. After exclusion, 224 732 patients were included in the final cohort. The results showed that compared with non-ADHD medication users, patients who were treated with MTH for more than 7 days had a similar risk of major CV events (HR 0.85, 95% CI 0.72 to 0.99; p=0.040). Identical trends were found in groups who were treated for more than 180 days (HR 0.83, 95% CI 0.69 to 1.00; p=0.050). The results of the sensitivity analyses were consistent with the main analyses across all groups and individual outcomes.

**Conclusion:**

Short-term MTH use did not increase CV risk among patients with ADHD. More evidence on long-term MTH use and risk of cardiogenic shock and death is warranted.

**WHAT IS ALREADY KNOWN ON THIS TOPIC**

- Cardiovascular (CV) safety has been a concern of attention deficit and hyperactivity disorder (ADHD) treatment. Although ADHD medication use does not appear to be associated with a significant increase in the risk of acute CV events, the long-term safety of methylphenidate has not been evaluated.

**WHAT THIS STUDY ADDS**

- The use of methylphenidate is not associated with an increased risk of CV events in children and youth treated with either short (≥7 days) or long-term (≥180 days) exposure, in an at least 3 years of follow-up.

**HOW THIS STUDY MIGHT AFFECT RESEARCH, PRACTICE OR POLICY**

- Methylphenidate treatment was not associated with long-term CV risk among patients with ADHD, indicating that it remains a safe choice for treating ADHD in children and adolescents.

## Introduction

 Attention deficit and hyperactivity disorder (ADHD) is the most common mental disorder in the paediatric population, affecting more than 7% of children worldwide.^1^ In Taiwan, the annual prevalence of ADHD in children under age 18 is 1.24%, and ADHD affects about 7.5% of school-aged children.^2^ ADHD management includes behavioural, pharmacological and educational interventions.^3^ Methylphenidate (MTH) is widely used to treat ADHD worldwide and is usually considered effective and safe.^3^,^6^ However, cardiovascular (CV) risk is a potential concern of MTH given that it is a weak central nervous system stimulant.

Multiple case studies have documented serious CV events among patients receiving ADHD medications,^7 8^ including sudden deaths, myocardial infarction, stroke and fatal arrhythmia. Previous studies have also indicated a potential link between MTH and a rise in blood pressure and heart rate,^9^,^14^ although these findings were not conclusive.^15 16^ With the reports highlighting the safety concerns associated with the treatment of ADHD, the US Food and Drug Administration (US FDA) deliberated on adding a black box warning to ADHD drugs concerning potential heart risks between 2006 and 2007.^17 18^

Later in 2011, the US FDA sponsored a large retrospective cohort study of ADHD medications including MTH, atomoxetine and other ADHD medications due to concerns about CV adverse effects. The study found no significant association between ADHD medication use and serious CV events.^19^ Nonetheless, there is a request for more comprehensive long-term safety assessments regarding MTH.^14 20^ Therefore, the aim of this study was to assess the association between MTH treatment and risk of CV events over a longer follow-up period than has been previously evaluated.

## Methods

### Database

Registry and claims data from the Health and Welfare Database, organised and maintained by the Health and Welfare Data Science Center, Ministry of Health, were used in this study. The National Health Insurance (NHI) programme was launched in 1995 and covers over 99% of the population in Taiwan. The NHI claims data contain information on patient demographics, diagnosis, procedures and prescription drugs. We linked birth certificate data to the administrative claims data at the individual level to identify patients’ conditions at birth. We also used the registry for catastrophic illness patients to identify children with congenital diseases or catastrophic illnesses. All data were deidentified and stored securely at the Health and Welfare Data Science Center.

### Study design and sample

This was a nationwide retrospective cohort study using data from 1 January 2003 to 31 December 2017. Patients aged 3–18 years, who were newly diagnosed with ADHD between 1 January 2006 and 31 December 2014, were enrolled in this study. The initiation of our enrolment period began in 2006 due to concerns with the data quality from 2003 to 2005, and all patients were required to have at least 1 year of data available for baseline assessment. The enrolment period ended in 2014 to ensure that all study subjects had at least 3 years of follow-up data. ADHD was defined by at least one inpatient or two outpatient diagnoses of ADHD in any position, identified by the International Classification of Diseases, Ninth Revision, Clinical Modification (ICD-9-CM) code 314.X and ICD-10-CM code F90. We excluded patients who were pregnant or diagnosed with cancer during the study period. Patients with multiple birthdate records, unknown sex, unknown insurance status, unknown date of first ADHD diagnosis or those who were treated with MTH or atomoxetine before their first ADHD diagnosis were also excluded. We identified 244 732 patients with ADHD aged 3–18 years between 2006 and 2014.

We next classified patients with ADHD into an MTH group, atomoxetine group and non-ADHD medication user group based on their first prescription after ADHD diagnosis. Because of the NHI reimbursement restriction, only a small proportion of patients with ADHD received atomoxetine as their initial treatment at the time of the study. Therefore, we focused our analyses on MTH. MTH users were further separated into two groups according to the duration of their initial therapy: ≥7 days and ≥180 days treatment groups to reflect short-term and long-term MTH treatment, respectively. The index date was the date when MTH users met the treatment definition (ie, day 7 and day 180). Patients were considered to have continuous drug treatment if the gap between two consecutive prescriptions was less than 90 days. For non-users, the index date was defined as 7 or 180 days after the first ADHD diagnosis between 2006 and 2014. We further excluded patients who had acute coronary syndrome (ACS), arrhythmia, stroke, cardiogenic shock or head injury within 12 months before the index date. Anatomical Therapeutic Chemical (ATC) classification system codes were used to identify the MTH (N06BA04) prescription.

Under the NHI programme, the ICD-9-CM codes were used to record diagnoses until the end of 2015, and the ICD-10-CM has been used since 2016. The ICD-9-CM codes used to identify the conditions for exclusion are listed in online supplemental appendix table 1.

### Patient involvement

Patients did not participate in formulating the research question, study design or the execution of the research. Patients were not involved in results interpretation and manuscript development. There are no intentions to share the research findings with study participants or the relevant patient community.

### Outcomes and follow-up

The studied outcomes included the following acute and severe CV events: ACS, arrhythmia, stroke, cardiogenic shock, cardiogenic shock and all-cause mortality (see online supplemental appendix table 2 for the ICD codes). All of these conditions were identified by primary diagnosis of inpatient admissions or emergency room visits. We combined all safety events as a composite outcome in the initial analysis and analysed each outcome separately in subsequent analyses. The intention-to-treat analysis approach was applied in the main analysis, in which patients were followed based on their initial group assignment until an event occurred or the end of the study (31 December 2017) regardless of subsequent treatment changes.

### Covariates

Potential confounders and CV risk factors were measured and adjusted in this study, including patients’ demographics, comorbidities and concomitant medications. Patients’ baseline comorbidities were identified in the 12 months before the index date, and concomitant medications were identified in the 3 months prior to the index date. Comorbidities were identified with at least one diagnosis of the following disease: liver disease, renal disease, thyroid disorders, epilepsy and psychiatric disorders (see online supplemental appendix table 3 for the ICD codes). Concomitant medication use was identified by the ATC codes (online supplemental appendix table 4). We also adjusted behaviour therapy using the NHI procedure codes, including occupational therapy and psychosocial rehabilitation therapy.

### Statistical analyses

First, we matched the MTH and atomoxetine users with non-users by age, sex and index date (±2 weeks). Next, one-to-one propensity score matching (PSM) with a calliper of width equal to 0.2 of the SD was performed to balance the baseline covariates between groups. The definition of no significant imbalance between groups was the absolute value of the standardised differences less than 0.1. Cox proportional hazard models were used to estimate CV risk for MTH and atomoxetine versus its matched non-user group. The covariates that were unbalanced after matching were further adjusted in the regression models. A p<0.05 was considered statistically significant. All statistical procedures were performed using SAS V.9.4 (SAS Institute).

### Sensitivity analyses

We performed several additional sensitivity analyses to check the robustness of our results. First, we adjusted the definition of the outcome by adding coronary artery disease, chronic heart failure and hypertensive disease to evaluate the risk of chronic CV disease. Second, to reduce potential bias among patients with congenital heart disease, for which physicians tend to prescribe non-medical treatment or low doses of medications, we identified patients who were diagnosed with both congenital heart disease and ADHD to perform subgroup analyses. Third, patients who had their first diagnosis and first prescription on the same day were selected to evaluate the risk, in order to reduce the potential effect of the gap between the first diagnosis to the first prescription.

## Results

### Baseline characteristics

**Figure 1:**
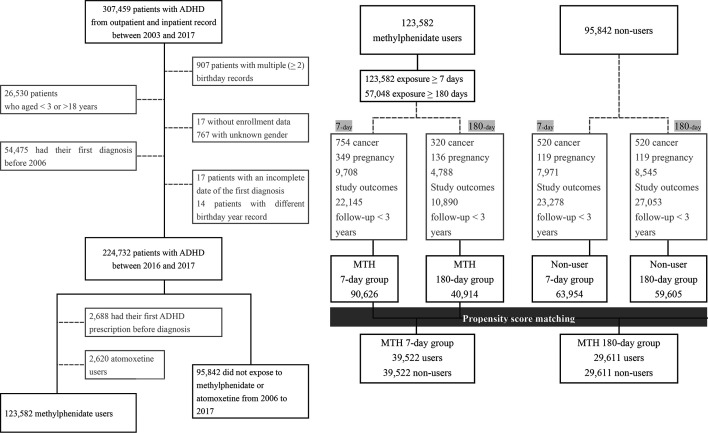
Sample selection flow chart. ADHD, attention deficit and hyperactivity disorder.

Figure 1 presents the sample selection flow chart. Starting from 244 732 patients with ADHD aged 3–18 years old, we further identified 123 582 patients receiving MTH and 95 842 patients without medication treatment. Among the MTH users, we identified 123 582 patients with at least 7 days exposure and 57 048 patients with at least 180 days exposure. After the PSM, the final study population was composed after PSM with 39 522 users paired with non-users in the 7-day MTH group, and 29 611 users paired with non-users in the 180-day MTH group.

Table 1 shows the baseline characteristics between MTH and atomoxetine users and non-users after PSM. Before matching (online supplemental appendix table 5), MTH users were older than non-users (8–9 years old vs 6–7 years old) and around 70%–80% were males among all four groups. The MTH users tended to have higher utilisation of antihypertensive (0.5%–0.6% vs 0.1%–0.2%) and antipsychotic (2-3% vs 1-2%) agents than non-users. Congenital heart disease was more common in non-users than in MTH users (7% vs 5%), but a higher proportion of non-users received behavioural treatment than MTH users (37% vs 5% in the 7-day MTH group; 9% vs 3% in the 180-day MTH group). After PSM, the absolute value of standardised differences was less than 0.1 for most of the covariates except anxiety and intellectual disability in the 7-day MTH group. Because of the privacy rules of the Health and Welfare Data Science Center, all cells that contained numbers less than 5 were substituted with ≤5.

**Table 1:** Baseline characteristics (after propensity score matching)

	Methylphenidate 7-day group			Methylphenidate 180-day group		
	Non-user	MTH	Standardised	Non-user	MTH	Standardised
	n=39 522	n=39 522	Difference	n=29 611	n=29 611	Difference
Age, mean (SD)	7.37 (3.14)	7.83 (2.73)	0.1574	8.64 (2.88)	8.86 (2.63)	0.078
Age group						
3–6	17 756 (44.93)	17 756 (44.93)	0	4480 (15.13)	4480 (15.13)	0
7–12	18 479 (46.76)	18 479 (46.76)		21 592 (72.92)	21 592 (72.92)	
≥13	3287 (8.32)	3287 (8.32)		3539 (11.95)	3539 (11.95)	
Sex, male	30 870 (78.11)	30 870 (78.11)	0	23 346 (78.84)	23 346 (78.84)	0
Comorbidities						
Hypertension	40 (0.10)	23 (0.06)	0.0152	30 (0.10)	28 (0.09)	0.002
Chronic heart failure	79 (0.20)	61 (0.15)	0.0108	31 (0.10)	37 (0.12)	0.006
Congenital heart disease	2509 (6.35)	2488 (6.30)	0.0022	1647 (5.56)	1726 (5.83)	0.012
Other heart disease	250 (0.63)	247 (0.62)	0.0010	161 (0.54)	179 (0.60)	0.008
Liver disease	134 (0.34)	107 (0.27)	0.0124	114 (0.38)	114 (0.38)	0
Renal disease	59 (0.15)	69 (0.17)	0.0063	46 (0.16)	41 (0.14)	0.004
Dialysis	<5	<5	0	<5	<5	0
Tic disorder	1427 (3.61)	1154 (2.92)	0.0389	1288 (4.35)	971 (3.28)	0.056
Intellectual disability	2344 (5.93)	3592 (9.09)	0.1200	2122 (7.17)	2207 (7.45)	0.011
Anxiety disorders	2559 (6.47)	4053 (10.26)	0.1369	2757 (9.31)	2924 (9.87)	0.019
Depressive disorders	611 (1.55)	541 (1.37)	0.0148	628 (2.12)	447 (1.51)	0.046
Thyroid disorder	134 (0.34)	134 (0.34)	0	116 (0.39)	114 (0.38)	0.001
Epilepsy	890 (2.25)	663 (1.68)	0.0414	725 (2.45)	530 (1.79)	0.046
Concomitant medications						
Aspirin	65 (0.16)	66 (0.17)	0.0006	31 (0.10)	37 (0.12)	0.006
Alpha/beta-blocker	99 (0.25)	59 (0.15)	0.0227	28 (0.09)	40 (0.14)	0.012
Antiarrhythmic agent	9 (0.02)	<5 (0.01)	0.0099	<5	<5	0.004
Antihypertensive agent	164 (0.41)	199 (0.50)	0.0131	65 (0.22)	92 (0.31)	0.018
Antihyperlipidaemic agent	18 (0.05)	20 (0.05)	0.0023	6 (0.02)	9 (0.03)	0.006
Calcium channel blockers	<5 (0.01)	<5 (0.01)	0.0027	<5	<5	0.008
Diuretics	16 (0.04)	14 (0.04)	0.0026	8 (0.03)	9 (0.03)	0.002
Nitrate	<5	<5	0	<5	<5	0
Vasodilators	<5	<5	0	<5	<5	0
Antiseizure agent	519 (1.31)	415 (1.05)	0.0244	217 (0.73)	217 (0.73)	0
Anxiolytic agent	791 (2.00)	715 (1.81)	0.0141	312 (1.05)	342 (1.15)	0.010
Bupropion	300 (0.76)	138 (0.35)	0.0552	41 (0.14)	40 (0.14)	0.001
SSRI/SSNRI	536 (1.36)	571 (1.44)	0.0075	77 (0.26)	161 (0.54)	0.045
Tricyclic antidepressant	306 (0.77)	381 (0.96)	0.0204	60 (0.20)	87 (0.29)	0.018
Antipsychotic agent	967 (2.45)	852 (2.16)	0.0194	363 (1.23)	414 (1.40)	0.015
Central acting sympathomimetic	<5	<5	0	<5	<5	0
Bronchodilator	708 (1.79)	718 (1.82)	0.0019	400 (1.35)	451 (1.52)	0.015
Xanthine derivate	2668 (6.75)	2928 (7.41)	0.0257	1657 (5.60)	1890 (6.38)	0.033
Non-medical treatment	4133 (10.46)	4251 (10.76)	0.0097	828 (2.80)	965 (3.26)	0.027
Median follow-up duration (years)	7.45	7.45		7.25	7.24	

MTHmethylphenidateSSRI/SSNRIselective serotonin reuptake inhibitor/serotonin and norepinephrine reuptake inhibitor

### Main analyses

**Figure 2:**
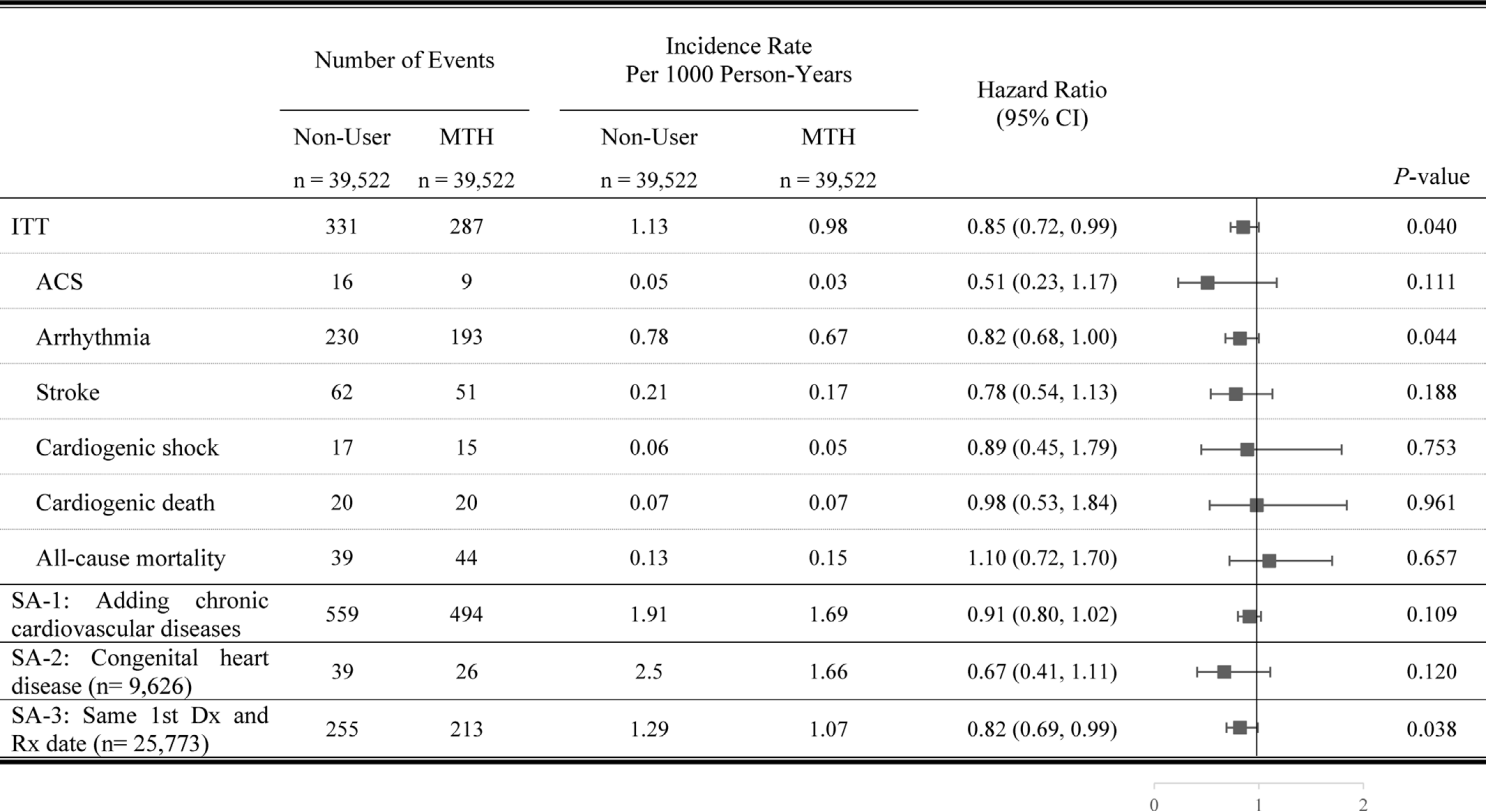
Main and Sensitivity Analyses of Outcome Among the Methylphenidate 7-Day Group.

Figure 2 presents the results from the 7-day MTH group. The median follow-up duration was 7.45 years for both the MTH users and non-users. The risk of composite outcome was lower in users than in non-users with marginal significance (adjusted HR 0.85, 95% CI 0.72 to 0.99; p=0.040). For individual outcomes, we found comparable risk between users and non-users for ACS regarding arrhythmia, stroke, cardiogenic shock, cardiogenic death and all-cause mortality.

**Figure 3:**
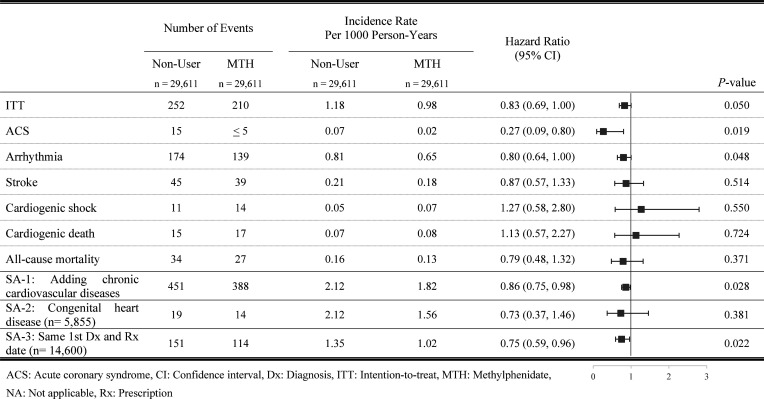
Main and Sensitivity Analyses of Outcome Among the Methylphenidate 180-Day Group.

Figure 3 shows the results of CV risk associated with MTH use in the 180-day MTH group compared with non-users. The median follow-up duration was 7.24 years for MTH users and 7.25 years for non-users. The results from the 180-day MTH group were generally consistent with the 7-day MTH group, but the overall risk of composite outcome was non-significant (HR 0.83, 95% CI 0.69 to 1.00; p=0.050). None of the individual outcomes were statistically significant.

### Sensitivity analysis 1: adding chronic events as the outcome

In the 7-day MTH group, the result of the composite outcome was identical to the main analysis, which revealed no significantly increased risk (HR 0.91, 95% CI 0.80 to 1.02; p=0.109) (figure 2). In the 180-day MTH group, the tendency was consistent with the main analysis in that the risk of composite outcome was significantly lower in the treatment group than in the non-user group (HR 0.86, 95% CI 0.75 to 0.98; p=0.028) (figure 3).

### Sensitivity analysis 2: children with congenital heart disease

We identified 9626 and 5855 patients who had a diagnosis of congenital heart disease in the 7-day and 180-day MTH groups, respectively. The results were consistent with those in the main analysis for both the 7-day and 180-day MTH groups.

### Sensitivity analysis 3: same date of the first diagnosis and prescription

From the final cohort, 35 936 MTH users who had their first diagnosis and first prescription on the same day were identified and included in this sensitivity analysis. After one-to-one PSM, there were 25 773 children in the 7-day MTH group and 14 600 children in the 180-day MTH group. The results were identical to the main analysis for both the 7-day and 180-day MTH groups.

## Discussion

Our study complements the evidence on MTH use and risk of composite serious CV outcomes in both the short-term and long-term use groups. Overall, the study results suggest that use of MTH is not associated with an increased risk of CV events in children and youth treated with MTH for more than 7 days, consistent with previous cohort studies in children and adolescents with ADHD.^13 21^ A case–control study, however, showed an association between psychostimulant use and sudden death, but the study was not focused on the ADHD population and treatment.^22^ Additionally, potential protopathic bias cannot be ruled out in that case–control study, as psychostimulant treatment was assessed right before sudden death. In our study, a tendency for higher all-cause death risk was found in the 7-day MTH group, but not in the 180-day group. One possible explanation was the depletion-of-susceptibles bias, where the risk of an acute event associated with drug use increases at the beginning of drug exposure and decreases after a longer duration of drug exposure. A similar trend was also found in a prior self-controlled case series study, in which the highest risk of arrhythmia was observed within 1–3 days after the initiation of MTH, and the risk gradually decreased over longer time periods.^23^

Our findings, while not statistically significant, indicated a trend towards a greater risk of cardiogenic shock and cardiogenic death in the 180-day MTH group. However, this potential risk increase was not observed in the 7-day MTH group, implying that long-term use of MTH may increase the risk of cardiogenic shock and death. Whether the CV risk associated with MTH use varies by treatment duration remains inconclusive and deserves further investigation.

Another noteworthy aspect of our study is the difference in baseline characteristics between the two groups. It appeared that a higher proportion of non-users had CV pre-existing conditions compared with MTH users, suggesting that patients’ CV medical history may influence prescribers to choose non-stimulant alternatives. However, given the standardised differences of the pre-existing CV conditions were not large, and MTH accounted for over 98% of the prescriptions at the time we conducted study,^24^ the potential selection bias in this study should be minimal.

To the best of our knowledge, this is one of the few retrospective studies to examine the association between MTH use and long-term CV risk. We retained at least 3 years of follow-up data in this cohort study. Several sensitivity analyses were performed to test the robustness of the study findings. We adjusted the definition of the study outcome to include chronic CV diseases, such as hypertensive disease and coronary artery disease, and the results suggested no significant increase in the risk of chronic CV diseases. In addition, we investigated the risk of long-term medication use in the 180-day group and performed a sensitivity analysis in a population with congenital heart disease. All of the results were consistent with the main findings.

Our study had several limitations. First, patients with ADHD were identified by diagnosis codes only, and the diagnosis was not validated by other instruments. We, therefore, required at least two outpatient diagnoses of ADHD diagnosis to avoid misclassification of the study population. Second, the database did not provide information on the severity of ADHD. We tried to eliminate the potential bias by adjusting for behavioural treatment as a covariate and performing PSM, but potential residual confounding by disease severity cannot be completely ruled out. Third, we only had prescribing and refill records, and the actual adherence level was unknown. Finally, patients with congenital heart disease have a higher risk of CV events and tend to use atomoxetine rather than MTH as pharmacotherapy for ADHD. Because of the small sample size, however, we were unable to perform further analyses on this group. A comprehensive comparison of the CV risk between MTH and atomoxetine among patients with congenital heart disease is required.

## Conclusion

Our study demonstrates that short-term use of MTH is not associated with a long-term risk of CV events and remains a safe choice for ADHD treatment in children and adolescents. Nevertheless, further investigations are needed regarding the potential risk of cardiogenic shock and death related to long-term use of MTH.

## supplementary material

10.1136/bmjpo-2024-002753 online supplemental file 1

## Data Availability

Data may be obtained from a third party and are not publicly available.
