# Supplementary material for: Association between methylphenidate use and long-term cardiovascular risk in paediatric patients with attention deficit and hyperactivity disorder
Source: BMJ Paediatr Open. 2024 Sep 3;8(1):e002753. doi: 10.1136/bmjpo-2024-002753 (PMC11428982; doi:10.1136/bmjpo-2024-002753)
Supplement: online supplemental file 1 [file bmjpo-8-1-s001.pdf]

**Appendix Table 1. Codes of the Exclusion Criteria**

| Disease | ICD-9-CM codes |
|---|---|
| **Cardiovascular disease** | |
| Acute coronary syndrome | 410, 411 |
| Arrhythmia | 427 |
| Cardiogenic shock | 785.51 |
| **Stroke event** | |
| Subarachnoid hemorrhage | 430 |
| Hemorrhage stroke | 431, 432 |
| Ischemic stroke | 433, 434, 436 |
| Head injury | 959.01 |
| **Other condition** | |
| Pregnant | V22 |
| Cancer | 140–239 |

## Appendix Table 2. ICD-9/10-CM Codes of the Studied Outcomes

| Disease | ICD-9-CM codes | ICD-10-CM codes |
|---|---|---|
| **Cardiovascular disease** | | |
| Acute coronary syndrome | 410, 411 | I20.0<br>I21.0, I21.01, I21.02, I21.19, I21.9<br>I22.0, I22.1<br>I24.0, I24.1, I24.8, |
| Arrhythmia | 427 | I46.2, 8, 9,<br>I47.0, 1, 2, 9<br>I48.0, 1, 2, 3, 4, 91, 92<br>I49.0, 01, 02, 1, 3, 40 |
| Cardiogenic shock | 785.51 | R57 |
| **Stroke event** | | |
| Subarachnoid hemorrhage | 430 | I60 |
| Hemorrhage stroke | 431, 432 | I61.0-6, 8, 9<br>I62.00-03, 1, 9 |
| Ischemic stroke | 433, 434, 436 | I63.02, 031, 032, 039, 1, 12, 131, 132, 139, 22, 231, 232, 239, 311, 312, 319, 321, 322, 329<br>I65.01, 02, 03, 21, 22, 23, 29<br>I66.01-03, 09, 11-13, 19, 21-23, 29, 3<br>I67.89 |
| **Mortality (listed code with death record)** | | |
| Cardiac death | 427.5, 99.60, 99.63 | I46.2, 8, 9 |
| All-cause death | 798 | R99 |

## Appendix Table 3. ICD-9-CM Codes of the Comorbidities

| Disease | ICD-9-CM codes |
|---|---|
| **Cardiovascular disease** | |
| Coronary artery disease | 413, 414 |
| Congestive heart failure | 428 |
| Hypertensive disease | 401-405 |
| Others | |
| Acute rheumatic fever | 390–392 |
| Chronic rheumatic heart disease | 393–398 |
| Acute pericarditis | 420 |
| Acute and subacute endocarditis | 421 |
| Acute myocarditis | 422 |
| Other diseases of pericardium | 423 |
| Other diseases of endocardium | 424 |
| Cardiomyopathy | 425 |
| Conduction disorders | 426 |
| Ill-defined descriptions and complications of heart disease | 429 |
| **Congenital heart disease** | |
| Bulbus cordis anomalies and anomalies of cardiac septal closure | 745 |
| Other congenital anomalies of heart | 746 |
| Other congenital anomalies of circulatory system | 747 |
| anomalies of cardiovascular system | 0300-0310 (NHI code) |
| **Liver disease** | |
| Chronic liver disease and cirrhosis | 571 |
| **Renal disease** | |
| Nephritis, Nephrotic Syndrome, And Nephrosis | 580–589 |
| **Psychiatric disorders** | |
| Tic disorder | 307.2 |
| Intellectual disability | 317–319 |
| Anxiety disorders | 300 |
| Depressive disorders | 296.2, 296.3, 300.4, 311 |
| **Others** | |
| Disorders Of Thyroid Gland | 240–246 |
| Epilepsy | 345 |

## Appendix Table 4. ATC Codes of the Concomitant Medications

| Medication | ATC codes |
| --- | --- |
| Aspirin | B01AC06 |
| Beta-blocker | C07 |
| Antiarrhythmic agent | C01B |
| Antihypertensive agent | C02, C09 |
| Antihyperlipidemic agent | C10 |
| Calcium channel blocker | C08 |
| Diuretics | C03 |
| Nitrate | C01DA |
| Vasodilators | C01DX |
| Antiseizure agent | N03A |
| Anxiolytic agent | N05B |
| Bupropion | N06AX12 |
| SSRI / SSNRI | N06AB |
| Tricyclic antidepressant | N06AA |
| Centrally acting sympathomimetic | N06BA |
| Antipsychotic agent | N05A |
| Bronchodilator | R03A |
| Xanthine derivate | C03BD01, R03DA |

SSRI/SSNRI: Selective serotonin reuptake inhibitor/serotonin and norepinephrine reuptake inhibitor.

**Appendix Table 5. Baseline Characteristics Among the Methylphenidate 7- and 180-Day Groups: Before Propensity Score Matching**

| | Methylphenidate 7-Day Group | | | Methylphenidate 180-Day Group | | |
|---|---|---|---|---|---|---|
| n, (%) | Non-User n = 63,954 | MTH n = 90,626 | Standardized Difference | Non-User n = 59,605 | MTH n = 40,914 | Standardized Difference |
| Age, mean (SD) | 6.42 (2.90) | 8.88 (2.89) | 0.8482 | 6.91 (2.92) | 9.15 (2.77) | 0.7872 |
| Age group | | | | | | |
| 3–6 | 39603 (61.92) | 19943 (22.01) | | 32063 (53.79) | 4858 (11.87) | |
| 7–12 | 21009 (32.85) | 57872 (63.86) | 0.8926 | 23738 (39.83) | 29675 (72.53) | 1.0035 |
| ≥13 | 3342 (5.23) | 12811 (14.14) | | 3804 (6.38) | 6381 (15.60) | |
| Sex, male | 46994 (73.48) | 72558 (80.06) | 0.8842 | 43774 (73.44) | 33200 (81.15) | 0.9992 |
| Comorbidities | | | | | | |
| Hypertension | 55 (0.09) | 70 (0.08) | 0.0031 | 55 (0.09) | 39 (0.10) | 0.0010 |
| Chronic heart failure | 139 (0.22) | 107 (0.12) | 0.0243 | 129 (0.22) | 42 (0.10) | 0.0285 |
| Congenital heart disease | 4706 (7.36) | 4620 (5.10) | 0.0936 | 4340 (7.28) | 2134 (5.22) | 0.0854 |
| Other heart disease | 394 (0.62) | 520 (0.57) | 0.0055 | 378 (0.63) | 231 (0.56) | 0.0090 |
| Liver disease | 188 (0.29) | 356 (0.39) | 0.0169 | 190 (0.32) | 179 (0.44) | 0.0193 |
| Renal disease | 115 (0.18) | 144 (0.16) | 0.0051 | 114 (0.19) | 51 (0.12) | 0.0168 |
| Dialysis | ≤ 5 | ≤ 5 | 0 | ≤ 5 | ≤ 5 | 0 |

MTH: Methylphenidate, SD: Standard deviation.

**Appendix Table 5. (Continued)**

| n, (%) | Methylphenidate 7-Day Group | | | Methylphenidate 180-Day Group | | |
|---|---|---|---|---|---|---|
| | Non-User n = 63,954 | MTH n = 90,626 | Standardized Difference | Non-User n = 59,605 | MTH n = 40,914 | Standardized Difference |
| Tic disorder | 1735 (2.71) | 2809 (3.10) | 0.0230 | 1788 (3.00) | 1394 (3.41) | 0.0231 |
| Intellectual disability | 2629 (4.11) | 7119 (7.86) | 0.1584 | 3073 (5.16) | 3626 (8.86) | 0.1456 |
| Anxiety disorders | 2643 (4.13) | 15182 (16.75) | 0.4217 | 3124 (5.24) | 8168 (19.96) | 0.4549 |
| Depressive disorders | 632 (0.99) | 1813 (2.00) | 0.0835 | 739 (1.24) | 785 (1.92) | 0.0545 |
| Thyroid disorder | 189 (0.30) | 322 (0.36) | 0.0105 | 180 (0.30) | 158 (0.39) | 0.0144 |
| Epilepsy | 1238 (1.94) | 1266 (1.40) | 0.0421 | 1339 (2.25) | 623 (1.52) | 0.0532 |
| Concomitant medications | | | | | | |
| Aspirin | 120 (0.19) | 126 (0.14) | 0.0120 | 90 (0.15) | 42 (0.10) | 0.0136 |
| Alpha / Beta-blocker | 112 (0.18) | 233 (0.26) | 0.0177 | 46 (0.08) | 65 (0.16) | 0.0238 |
| Antiarrhythmic agent | 13 (0.02) | 13 (0.01) | 0.0045 | 6 (0.01) | 6 (0.01) | 0.0041 |
| Antihypertensive agent | 175 (0.27) | 486 (0.54) | 0.0414 | 88 (0.15) | 269 (0.66) | 0.0806 |
| Antihyperlipidemic agent | 36 (0.06) | 37 (0.04) | 0.0070 | 36 (0.06) | 10 (0.02) | 0.0175 |
| Calcium channel blockers | ≤ 5 (0.01) | 10 (0.01) | 0.0051 | ≤ 5 | ≤ 5 | 0.0063 |
| Diuretics | 21 (0.03) | 37 (0.04) | 0.0042 | 18 (0.03) | 10 (0.02) | 0.0035 |

MTH: Methylphenidate

**Appendix Table 5. (Continued)**

| n, (%) | Methylphenidate 7-Day Group | | | Methylphenidate 180-Day Group | | |
|---|---|---|---|---|---|---|
| | **Non-User n = 63,954** | **MTH n = 90,626** | **Standardized Difference** | **Non-User n = 59,605** | **MTH n = 40,914** | **Standardized Difference** |
| Nitrate | ≤ 5 | ≤ 5 | 0.0042 | ≤ 5 | ≤ 5 | 0.0017 |
| Vasodilators | ≤ 5 | ≤ 5 | 0 | ≤ 5 | ≤ 5 | 0 |
| Anti-seizure agent | 694 (1.09) | 915 (1.01) | 0.0074 | 365 (0.61) | 294 (0.72) | 0.0131 |
| Anxiolytic agent | 1103 (1.72) | 1842 (2.03) | 0.0227 | 608 (1.02) | 534 (1.31) | 0.0266 |
| Bupropion | 313 (0.49) | 438 (0.48) | 0.0009 | 50 (0.08) | 76 (0.19) | 0.0278 |
| SSRI/SSNRI | 541 (0.85) | 1828 (2.02) | 0.0987 | 109 (0.18) | 354 (0.87) | 0.0946 |
| Tricyclic antidepressant | 326 (0.51) | 815 (0.90) | 0.0466 | 77 (0.13) | 230 (0.56) | 0.0738 |
| Antipsychotic agent | 1162 (1.82) | 2276 (2.51) | 0.0477 | 492 (0.83) | 797 (1.95) | 0.0961 |
| Central acting sympathomimetic | ≤ 5 | ≤ 5 | 0.0047 | ≤ 5 | ≤ 5 | 0 |
| Bronchodilator | 1505 (2.35) | 1281 (1.41) | 0.0692 | 1407 (2.36) | 541 (1.32) | 0.0773 |
| Xanthine derivate | 5234 (8.18) | 5609 (6.19) | 0.0773 | 4622 (7.75) | 2430 (5.94) | 0.0719 |
| Non-medical treatment | 23698 (37.05) | 4408 (4.86) | 0.8611 | 5411 (9.08) | 1028 (2.51) | 0.2838 |

MTH: Methylphenidate
