# Supplementary material for: Association between methylphenidate use and long-term cardiovascular risk in paediatric patients with attention deficit and hyperactivity disorder
Source: BMJ Paediatr Open. 2024 Sep 3;8(1):e002753. doi: 10.1136/bmjpo-2024-002753 (PMC11428982; doi:10.1136/bmjpo-2024-002753)
Supplement: Supplementary file 2 [file bmjpo-2024-002753.reviewer_comments.pdf]

PEER REVIEW HISTORY

BMJ Paediatrics Open publishes all reviews undertaken for accepted manuscripts. Reviewers are asked to complete a checklist review form and are provided with free text boxes to elaborate on their assessment. These free text comments are reproduced below.

ARTICLE DETAILS

**TITLE (PROVISIONAL)**	Association between methylphenidate use and long-term cardiovascular risk in pediatric patients with attention deficit and hyperactivity disorder.
**AUTHORS**	Liao, Heng-Ching<br>Hsu, Chien-Ning<br>Lin, Fang-Ju<br>Gau, Susan Shur-Fen<br>WANG, CHI-CHUAN

VERSION 1 - REVIEW

**REVIEWER NAME**	Dr. Huang Chuan-Chin
**REVIEWER AFFILIATION**	Brigham and Women's Hospital<br>Medicine<br>75 Francise street<br>Boston<br>Massachusetts<br>02115-6195<br>United States
**REVIEWER CONFLICT OF INTEREST**	No
**DATE REVIEW RETURNED**	26-Jun-2024

**GENERAL COMMENTS**	In general, this study is well-done, providing evidence that MTH may not increase CV risk among ADHD. Here are some suggestions.<br><br>1. The authors should consider presenting the numerical values of the main findings in the abstract.<br>2. The results of SA-2 and SA-3 should be presented in separate tables since they have different sample sizes.<br>3. The use of a robust variance estimator to account for the matching nature of PSM is technically necessary. The authors may want to consider using robust variance estimators to account for clustering within matched sets. https://www.ncbi.nlm.nih.gov/pmc/articles/PMC4285179/#b38<br>While this concern might be major in other studies, it is minor in this study since the results favor a null finding. Therefore, I would make this an optional choice for the authors.<br>4. Considering the low incidence of individual outcome measures and some pre-existing comorbidities remaining not well-unbalanced after PSM matching, it may make sense to perform a sensitivity analysis excluding participants with pre-existing comorbidities relevant to cardiovascular outcomes, such as hypertension, chronic heart failure, or congenital heart disease, to see whether MTH use increases cardiovascular risks in a population without pre-existing risks.<br>5. In Table 3, although not statistically significant, considerable adverse effect sizes were observed in cardiogenic shock and death. Given that similar observations were not found in the 7-day use setting, it may be not appropriate to conclude null findings for these two outcomes in the 180-

	day setting. Therefore, the authors may want to emphasize whether long-term use of MTH increases the risk of cardiogenic shock and death remains inconclusive.

**REVIEWER NAME**	Dr. Cesar A. Soutullo-Esperon
**REVIEWER AFFILIATION**	The University of Texas Health Science Center at Houston<br>Psychiatry and Behavioral Health<br>1941 East Road, Suite 2118<br>Houston<br>Houston<br>Texas<br>77054<br>United States
**REVIEWER CONFLICT OF INTEREST**	NO
**DATE REVIEW RETURNED**	23-Jul-2024

**GENERAL COMMENTS**	This is a very interesting paper, with nice methodology, and a very large sample of Children with ADHD treated with medications, both stimulants and non/-stimulants.<br>The issue about Cardiovascular safety of ADHD medication keeps coming back, and it is worth having more data that documents if these medications are indeed safe, with big cohorts and longer follow up periods.<br>I have some suggestions for the authors.<br>Introduction:<br>This paper is short and that is helpful, but it may be nice to review a bit more of the papers who indicated a safety concern, and the ones that documented safety, and why some may be positive for the association of MPH treatment and cardiovascular events, as at times there may be confounding variables.<br>There is a paper by Cooper WO in N Engl J of Medicine 2011 that documented no elevated CV risk with stimulants that may be worth adding to the literature review.<br>I think is important to educate the reader on what areas they need to check on a paper that is reporting a safety concern, and what variables have to be controlled for.<br><br>This paper does a good job also on documenting prior CV history that apparently was more frequent on non-MPH users. They may want to comment that maybe the presence of this history may have made the prescribers prefer to use alternatives to stimulants.<br><br>Methods<br>I think it would be useful to mention the N of 244,732 early in the Method section, for orientation purposes. It may also be helpful to report the mean time of exposure to stimulants and non-stimulants, and the mean follow-up period for those patients not on medication. Authors use the 7-day and 180-day mark, but I am not sure if those are the only time points they analyzed.<br><br>In page 9, results, baseline characteristics, authors refer to Table 5. However, it may be nice to have the % of antipsychotic utilization, anti-hypertensive agents, congenital heart disease etc in MTH users vs non users and the p to indicate significance. This will avoid the reader having to look for Table 5 ang bo back and forth to and from the text.<br><br>Also, since this paper is on CV risks, I suggest authors use the

	pharmacological term alpha-2 agonists if they are refering to clonidine and guanfacine for the treatment of ADHD when they speak about "antihypertensives", otherwise is a bit confsing.<br><br>Finally, the title is misleading and suggests there is an association of MPH and CV risk, I suggest to change to the more accurate:<br>Short- and long-term use of methylphanidate in nos associated with long term risk of cardiovascular events.<br>Or<br>methylphenidate was not associated with long term cardivascular events, both after short- and long-term use.<br><br>References: please add for a comprehensive review of the topic:<br>Faraone et al., 2021 The World Federation of ADHD International Consensus Statement: 208 Evidence-based conclusions about the disorder https://pubmed.ncbi.nlm.nih.gov/33549739/<br><br>Tables: all should be self explanatory, please add the total N.<br>Table 1. Baseline characteristics of 244,732 patients ages 3 t o 18 treated with stimulants, non-stimulants or no treatment for a mean duration of X years.<br><br>Table 2. Cardivascular adverse events in X patients treated with MPH for 7 days<br>Table 3, same thing but 180-days<br>Figuer 1, same details so one does not have to look for the n and the diagnosis and treated vs non treated in another page<br>Appendix Table 5, add the N there

VERSION 1 – AUTHOR RESPONSE

Reviewer: 1
Dr. Huang Chuan-Chin, Brigham and Women's Hospital

Comments to the Author
In general, this study is well-done, providing evidence that MTH may not increase CV risk among ADHD. Here are some suggestions.

1. **The authors should consider presenting the numerical values of the main findings in the abstract.**

   Response: Thank you for your suggestion. We have added the numerical values of the main findings in the abstract.

   Abstract (Pages 2-3, Lines 23-25)
   "……, patients who were treated with MTH for more than 7 days had a similar risk of major CV events (HR: 0.85, 95% CI: 0.75–0.99; *P* = 0.040). Identical trends were found in groups who were treated for more than 180 days (HR: 0.83, 95% CI: 0.69–1.00; *P* = 0.050)."

2. **The results of SA-2 and SA-3 should be presented in separate tables since they have different sample sizes.**

   Response: Thank you for your comment. We have added the sample size of SA-2 and SA-3 in Table 2 and Table 3 to further clarify that the sample sizes of these two sensitivity analyses were different from the sample size of the main analysis and SA-1. To facilitate easy comparison between the results of the main analysis and the sensitivity analyses for readers, we have

retained the results of SA-2 and SA-3 in the same result tables as the main analysis and SA-1 results.

3. **The use of a robust variance estimator to account for the matching nature of PSM is technically necessary. The authors may want to consider using robust variance estimators to account for clustering within matched sets.** https://www.ncbi.nlm.nih.gov/pmc/articles/PMC4285179/#b38 **While this concern might be major in other studies, it is minor in this study since the results favor a null finding. Therefore, I would make this an optional choice for the authors.**

   Response: We thank the reviewer for this insightful comment. We agree with the reviewer that it is necessary to use robust variance to account for the matching nature of PSM. Unfortunately, we were unable to perform further analysis because our data usage period has expired. As the reviewer mentioned, we expect to find similar results from the analysis with a robust variance estimator since we had a null finding in our study. As a result, our study conclusion will remain the same even if a robust variance estimator is used.

4. **Considering the low incidence of individual outcome measures and some pre-existing comorbidities remaining not well-unbalanced after PSM matching, it may make sense to perform a sensitivity analysis excluding participants with pre-existing comorbidities relevant to cardiovascular outcomes, such as hypertension, chronic heart failure, or congenital heart disease, to see whether MTH use increases cardiovascular risks in a population without pre-existing risks.**

   Response: We thank the reviewer for this valuable comment. Indeed, we considered performing subgroup or sensitivity analyses for patients without pre-existing cardiovascular conditions. However, we decided to focus on evaluating the cardiovascular risk among patients with congenital heart disease instead. This decision was based on the null findings of the main analysis, which suggested minimal risk increases even among patients with pre-existing conditions. Therefore, the risk increases would likely be even lower among patients without pre-existing conditions. It is more clinically meaningful to assess the potential cardiovascular risk increases among patients with pre-existing conditions. Consequently, we selected patients with congenital heart disease, a condition relevant to pediatric populations, for further analysis.

5. **In Table 3, although not statistically significant, considerable adverse effect sizes were observed in cardiogenic shock and death. Given that similar observations were not found in the 7-day use setting, it may be not appropriate to conclude null findings for these two outcomes in the 180-day setting. Therefore, the authors may want to emphasize whether long-term use of MTH increases the risk of cardiogenic shock and death remains inconclusive.**

   Response: Thank you for this insightful suggestion. A new paragraph has been incorporated into the Discussion section and the conclusions have been revised to reflect this point.

   Abstract, Conclusion (Page 3, Lines 29-30)
   "Short-term MTH use did not increase CV risk among ADHD patients. More evidence on long-term MTH use and risk of cardiogenic shock and death is warranted."

   Discussion (Pages 12-13, Lines 238-243)
   "Our findings, while not statistically significant, indicated a trend towards a greater risk of cardiogenic shock and cardiogenic death in the 180-day MTH group. However, this potential risk increase was not observed in the 7-day MTH group, implying that long-term use of MTH may increase the risk of cardiogenic shock and death. Whether the CV risk associated with MTH use varies by treatment duration remains inconclusive and deserves further investigation."

   Conclusion (Page 14, Lines 274-277)

“Our study demonstrates that short-term use of MTH is not associated with a long-term risk of CV events and remains a safe choice for ADHD treatment in children and adolescents. Nevertheless, further investigations are needed regarding the potential risk of cardiogenic shock and death related to long-term use of MTH.”

Reviewer: 2
Dr. Cesar A. Soutullo-Esperon, The University of Texas Health Science Center at Houston

Comments to the Author
This is a very interesting paper, with nice methodology, and a very large sample of Children with ADHD treated with medications, both stimulants and non/-stimulants.
The issue about Cardiovascular safety of ADHD medication keeps coming back, and it is worth having more data that documents if these medications are indeed safe, with big cohorts and longer follow up periods.

I have some suggestions for the authors.
Introduction:

1. **This paper is short and that is helpful, but it may be nice to review a bit more of the papers who indicated a safety concern, and the ones that documented safety, and why some may be positive for the association of MPH treatment and cardiovascular events, as at times there may be confounding variables.**
2. **I think is important to educate the reader on what areas they need to check on a paper that is reporting a safety concern, and what variables have to be controlled for.**

Response: We thank the reviewer for this suggestion. The Introduction has been updated to more accurately summarize research showing a positive association between MPH and CV risk along with studies reporting no findings, emphasizing the potential origins of CV risk concerns.

Introduction (Page 5, Lines 57-70)
"Multiple case studies have documented serious cardiovascular events among patients receiving ADHD medications,7 8 including sudden deaths, myocardial infarction, stroke, fatal arrhythmia. Previous studies have also indicated a potential link between MTH and a rise in blood pressure and heart rate, although these findings were not conclusive. 16 With the reports highlighting the safety concerns associated with the treatment of ADHD, the United States Food and Drug Administration (U.S. FDA) deliberated on adding a black box warning to ADHD drugs concerning potential heart risks between 2006 and 2007.

Later in 2011, the U.S. FDA sponsored a large retrospective cohort study of ADHD medications including MTH, atomoxetine, and other ADHD medications due to concerns of CV adverse effects. The study found no significant association between ADHD medication use and serious CV events. Nonetheless, there is a request for more comprehensive long-term safety assessments regarding MTH. Therefore, the aim of this study was to assess the association between MTH treatment and risk of CV events over a longer follow-up period than has been previously evaluated."

3. **There is a paper by Cooper WO in N Engl J of Medicine 2011 that documented no elevated CV risk with stimulants that may be worth adding to the literature review.**

Response: Thank you for your comment. This paper was included in the original draft and cited as reference 19.

4. **This paper does a good job also on documenting prior CV history that apparently was more frequent on non-MPH users. They may want to comment that maybe the presence of this history may have made the prescribers prefer to use alternatives to stimulants.**

Response: Thank you for raising this valuable point. We have added a paragraph in the Discussion section to address this point.

Discussion (Page 13, Lines 244-250)
"Another noteworthy aspect of our study is the difference in baseline characteristics between the two groups. It appeared that a higher proportion of non-users had CV pre-existing

conditions compared to MTH users, suggesting that patients' CV medical history may influence prescribers to choose non-stimulant alternatives. However, given the standardized differences of the pre-existing CV conditions were not large, and MTH accounted for over 98% of the prescriptions at the time we conducted study, the potential selection bias in this study should be minimal."

Methods

1. **I think it would be useful to mention the N of 244,732 early in the Method section, for orientation purposes. It may also be helpful to report the mean time of exposure to stimulants and non-stimulants, and the mean follow-up period for those patients not on medication. Authors use the 7-day and 180-day mark, but I am not sure if those are the only time points they analyzed.**

   Response: We thank the reviewer for these suggestions. A sentence was added in the Method section to report the N of 244,732. Regarding the exposure, we constructed two groups of samples; one sample consisted of patients using MTH for at least 7 days (follow-up started on day 8), and the other sample contained patients with at least 180 days of MTH exposure (follow-up started on day 181). These are the only two landmark points that we analyzed in this study. We have also reported the median follow-up duration in the Results section.

   Methods (Page 7, Lines 97-98)
   "We identified 244,732 patients with ADHD aged 3 to 18 years between 2006 and 2014."

   Results (Pages 10-11, Lines 191-192)
   "The median follow-up duration was 7.45 years for both the MTH users and non-users."

   Results (Page 11, Lines 198-199)
   "The median follow-up duration was 7.24 years for MTH users and 7.25 years for non-MTH users."

2. **In page 9, results, baseline characteristics, authors refer to Table 5. However, it may be nice to have the % of antipsychotic utilization, anti-hypertensive agents, congenital heart disease etc in MTH users vs non users and the p to indicate significance. This will avoid the reader having to look for Table 5 ang bo back and forth to and from the text.**

Response: Thank you for your suggestion. We have included the percentages of these baseline characteristics in the text.

Results (Page 10, Lines 178-184)
"Before matching (Appendix Table 5), both MTH users were older than non-users (8-9 years old versus 6-7 years old) and around 70-80% were males among all four groups. The MTH users tended to have higher utilization of anti-hypertensive (0.5-0.6% versus 0.1-0.2%) and anti-psychotic (2% versus 1%) agents than non-users. Congenital heart disease was more common in non-users than in MTH users (7% versus 5%), but a higher proportion of non-users received behavioral treatment than MTH users (37% versus 5% in the 7-day MTH group; 9% versus 3% in the 180-day MTH group)."

3. **Also, since this paper is on CV risks, I suggest authors use the pharmacological term alpha-2 agonists if they are refering to clonidine and guanfacine for the treatment of ADHD when they speak about "antihypertensives", otherwise is a bit confsing.**

Response: We thank the reviewer for this valuable point. In this study, we did not specifically measure clonidine and guanfacine for the treatment of ADHD but instead measure all types of antihypertensive agents. Since hypertension is a risk factor for CV events, baseline antihypertensive use is an important factor to be captured and adjusted as it indicates both the underlying condition (i.e., hypertension) and treatment.

4. **Finally, the title is misleading and suggests there is an association of MPH and CV risk, I suggest to change to the more accurate:**
   Short- and long-term use of methylphanidate in nos associated with long term risk of cardiovascular events. Or methylphenidate was not associated with long term cardivascular events, both after short- and long-term use.

   Response: We greatly appreciate your recommendation. Nonetheless, taking into account the feedback from Reviewer 1, there appears to be an increased risk of cardiogenic shock and mortality in the 180-day MTH group but not within the 7-day MTH group. Consequently, we have incorporated a new point of discussion and have altered the study's conclusion to state, "Our study demonstrates that short-term use of MTH is not associated with a long-term risk of CV events and remains a safe choice for ADHD treatment in children and adolescents. Nevertheless, further investigations are needed regarding the potential risk of cardiogenic shock and death related to long-term use of MTH."

   Given these revisions and the updated interpretation of our study's findings, we believe the current title more accurately represents the content of our research.

References

1. **please add for a comprehensive review of the topic:**
   Faraone et al., 2021 The World Federation of ADHD International Consensus Statement: 208 Evidence-based conclusions about the disorder
   https://pubmed.ncbi.nlm.nih.gov/33549739/

   Response: Thank you for your suggestion. We have cited the paper (Ref. 6) in the manuscript.

Tables and Figures

1. **all should be self explanatory, please add the total N.**

   Response: Thank you for the comment. Total Ns of SA-2 and SA-3 were added in Table 2 and Table 3.

2. **Table 1. Baseline characteristics of 244,732 patients ages 3 t o 18 treated with stimulants, non-stimulants or no treatment for a mean duration of X years.**

   Response: The number 244,732 was not the final sample for analysis. Rather, it was the initial count of patients identified with ADHD, and additional inclusion and exclusion criteria were implemented to establish the final sample for analysis (Figure 1). The numbers presented in Table 1 was the final samples for analysis, which shows the number of patients receiving MTH and no treatment. We have added the median follow-up durations in Table 1 according to the reviewer's suggestion.

3. **Table 2. Cardivascular adverse events in X patients treated with MPH for 7 days**
   Table 3, same thing but 180-days

   Response: The sample size of the 7-day (n= 39,522) and 180-day (n= 29,611) groups are presented in the heading of columns for Table 2 and Table 3. We have added the sample sizes for SA-2 and SA-3 as the number of patients included in these two sensitivity analyses were different from that of the main analysis.

4. **Figuer 1, same details so one does not have to look for the n and the diagnosis and treated vs non treated in another page**

Response: We appreciate the reviewer's suggestion. We have added more details about the sample selection process in the Methods and Results sections, and Figure 1 has been revised for better clarity.

Methods (Page 6, Lines 85-89)

"This was nationwide retrospective cohort study using data from January 1, 2003 to December 31, 2017. Patients aged 3 to 18 years, who were newly diagnosed with ADHD between January 1, 2006 and December 31, 2014, were enrolled in this study. The initiation of our enrollment period began in 2006 due to concerns with the data quality from 2003 to 2005, and all patients were required to have at least one year of data available for baseline assessment."

Results (Page 10, Lines 170-176)

"Figure 1 presents the sample selection flow chart. Starting from 244,732 patients with ADHD aged 3 to 18 years old, we further identified 123,582 patients receiving MTH and 95,842 patients without medication treatment. Among the MTH users, we identified 123,582 patients with at least 7 days exposure and 57,048 patients with at least 180 days exposure. After the propensity score matching, the final study population was composed after PSM with 39,522 users paired to non-users in the 7-day MTH group, and 29,611 users paired to non-users in the 180-day MTH group."

5. **Appendix Table 5, add the N there**

Response: The sample size of each group is presented in the heading of columns for Appendix Table 5. For the 7-day group, the n is 63,954 for non-users and 90,926 for MTH users; for the 180-day group, the n is 59,605 for non-users and 40,914 for MTH users.
